# LncRNA MEG3/CTCF-CXCR4 axis functions in the regulation of breast cancer cell migration

**DOI:** 10.1016/j.ncrna.2025.05.014

**Published:** 2025-05-28

**Authors:** Gusai Elhassan, Xiangxue Bu, Jiaxin Liu, Shuai Hou, Jinsong Yan, Haixin Lei

**Affiliations:** a Institute of Cancer Stem Cell, Cancer Center, Dalian Medical University, Dalian, China; b Department of Molecular Biology, Faculty of Medical Laboratory Sciences, Al Neelain University, Khartoum, Sudan; c Department of Hematology, Liaoning Medical Center for Hematopoietic Stem Cell Trans-plantation, The Second Hospital of Dalian Medical University, Dalian, China

**Keywords:** lncRNA MEG3, Breast cancer, Cell migration, CXCR4, CTCF

## Abstract

Loss or decreased expression of lncRNA MEG3 is a frequent event in the progression of many different malignancies. Overexpression of MEG3 in breast cancer cell lines MCF7 or MDA-MB-231 prevented cell migration, whereas depletion of MEG3 in human mammary epithelial cell line MCF10A strikingly promoted cell migration. As RNA-protein interactions are vital for RNA to function, RNP assembled on MEG3 *in vivo* was purified using affinity purification followed by mass spectrometry, which revealed ∼600 proteins with the potential to interact with MEG3. Bioinformatic analysis on RNA-seq data from MCF7 with MEG3 overexpression and MCF10A with MEG3 depletion led to the identification of CXCR4 as the major downstream mediator negatively regulated by MEG3 that facilitated breast cancer cell migration. In addition, the chromatin regulator CTCF emerged as the MEG3-binding protein that might regulate CXCR4 expression after comparison of proteins presenting in MEG3 lncRNP to ChIP-seq data and GPSAdb data of CXCR4. Further evidence was provided to show CTCF upregulated the expression of CXCR4 at transcriptional level, whereas co-expression of MEG3 with CTCF abolished transcriptional activation of CXCR4. Overall, our study pinpoints the importance of MEG3/CTCF-CXCR4 axis in regulating migration of breast cancer cells and provides novel insight into the mechanism of lncRNA MEG3 in cancer development.

## Highlights

• Mass spectrometry analysis reveals ?600 proteins in MEG3 lncRNP assembled in vivo.
• CXCR4 is the major downstream mediator negatively regulated by MEG3 that facilitates breast cancer cell migration.
• LncRNA MEG3 interacts with chromatin regulator CTCF to inhibit CXCR4 expression at transcriptional level.

## Abbreviations:

LncRNA: Long non-coding RNA
LncRNP: Long non-coding ribonucleoprotein
snRNP: Small nuclear ribonucleoprotein
RIP RNA: Immunoprecipitation.
FISH: Fluorescence in situ hybridization
DOX: Doxycycline

## 1 Introduction

With the identification of large number of long non-coding RNAs (lncRNAs), research interests have been focused on their potential roles in physiological and pathological conditions. Among them, maternally expressed gene 3 (MEG3) emerged as one of the frequently deregulated lncRNAs that played a vital role in cancer development.

The human MEG3 gene localizes in chromosome 14q32.3 [1] with the genomic sequence spans ∼35 kb, the representative transcript contains seven exons of 1592 nt [2]. MEG3 is a lncRNA with a typical nuclear localization, as assayed at both endogenous and exogenous levels [2,3]. Further investigation reveals that MEG3 contains a powerful nuclear retention element which interacts with U1 snRNP components to restrain MEG3 in the nucleus [2].

Decreased expression of lncRNA MEG3 has been reported in many malignancies[[4], [5], [6]], including breast cancer [7]. MEG3 expression has been found to correlate with the prognosis of cancer patients and the pathological grade of malignancy [4]. Furthermore, the low expression level of MEG3 has been correlated with poor prognosis in cervical cancer, meningioma, gastric cancer, colorectal cancer, and breast cancer[[8], [9], [10], [11], [12]]. In addition, decreased MEG3 expression in breast cancer and colorectal cancer was strongly correlated with TNM stage and lymph node metastasis [11,12].

Loss of MEG3 expression in tumors can result from mechanisms including gene deletion, promoter hypermethylation, and hypermethylation of intergenic differentially methylated region[[13], [14], [15], [16], [17], [18]], which disrupt regulatory mechanisms that control MEG3 expression levels [5]. In addition, MYC has been reported to regulate the expression of several lncRNAs including MEG3 in breast cancer cells, with MYC overexpression enhancing MEG3 level and inhibition of MYC decreasing MEG3 expression [19]. Whereas Wilms’ tumor 1 has been shown to enhance MEG3 expression transcriptionally in acute myeloid leukemia [20].

Lower expression of MEG3 has been reported to have major impacts on cell proliferation, apoptosis, and EMT in cancer development[[21], [22], [23], [24]]. Accumulated evidence supported that MEG3 was a tumor suppressor and its function could be mediated either by p53-dependent or p53-independent mechanisms. MEG3 overexpression led to substantial p53 stabilization and accumulation by reducing MDM2, the major regulator on the degradation of p53 protein [25]. Increased p53 level transcriptionally regulated downstream effectors including p21, Maspin, and KAI1 to inhibit cell proliferation, colony formation, migration, and invasion of breast cancer cells [26]. Interestingly, NF-κB signaling was reported to be required for MEG3-induced p53 activation in breast cancer cells [7]. MEG3 could also enhance p53 binding to the promoter of GDF15 which increased GDF15 expression [25].

The presence of p53-independent pathway was supported by the observations that MEG3 had similar effects in p53-null breast cancer cell line [26], and it could inhibit cell growth in cells lacking functional p53 [5,25]. Deletion of MEG3 in TNBC cell line revealed increased levels of TGF-β and N-cadherin as well as decreased expression of genes involved in extracellular matrix degradation and invasion, like MMP2, ZEB1, and COL3A1 [22]. In prostate cancer tissues and PC3 cells, MEG3 was found to interact with EZH2 to suppress the expression of EN2, a transcription factor functioning in cell proliferation and migration [24]. In retinoblastoma, MEG3 controlled proliferation and promoted apoptosis by impairing the activity of Wnt/β-catenin pathway [27]. Whereas in cervical cancer, MEG3 was reported to promote apoptosis and inhibit cell proliferation via binding to p-STAT3, which resulted in the ubiquitination and degradation of STAT3 [28].

In addition, MEG3 played a crucial role in epigenetic regulation by interacting with chromatin. MEG3 was shown to share common target genes like TGF-β with PRC2 complex component EZH2 [29]. Further investigation revealed the GA-rich sequence motifs enriched in MEG3-bound genomic regions functioned in targeting MEG3 RNA to chromatin via the formation of RNA-DNA triplexes [29]. Moreover, MEG3 could interact with JARID2 to have an impact on histone methylation and gene expression related to EMT [30].

Although previous studies provided insights into how MEG3 might function in the progression of cancer, most of them failed to explore that RNA-protein interactions were fundamental for RNA to function. Thus, in this study, we took the advantage of affinity purification based on MS2 hairpin and MS2-MBP fusion protein to identify proteins bound on MEG3 by mass spectrometry after purification of the lncRNP assembled on MEG3 *in vivo* [31], and provided further data to support that the MEG3/CTCF-CXCR4 axis as a novel mechanism for MEG3 in modulating cell migration of breast cancer.

## 2 Materials and methods

### 2.1 Constructs and antibodies

To generate MEG3 overexpression plasmids, MEG3 cDNA of transcript 1 was inserted in pLVX-TRE3G (Clontech, USA), pcDNA5/FRT/TO vector (Invitrogen), and modified pcDNA3.1 vector as described [2]. To generate CTCF overexpression plasmids, CTCF cDNA of transcript 1 was cloned in pcDNA3.1 with or without EGFP tag at C-terminal. All constructs were sequenced for confirmation.

Antibodies targeted to CTCF (Proteintech, 30428-1-AP, 1:1000), GAPDH (Proteintech, 10494-1-AP, 1:10000), MYH9 (Proteintech, 14844-1-AP, 1:500), PES1 (Proteintech, 13553-1-AP, 1:1000), IKBIP (Proteintech, 14589-1-AP, 1:500), PHB (Proteintech, 10787-1-AP, 1:1000) and CXCR4 (ABclonal, A19035, 1:1000) were used in Western blot.

### 2.2 Cell culture and transfection

MCF10A was cultured in MEGM™ (LONZA), MDA-MB-231 was cultured in RPMI 1640 (Gibco), MCF7, HeLa and FRT-HeLa cell lines were cultured in DMEM (Gibco), all supplemented with 10 % FBS (VivaCell, China).

For transient transfection, the cells were cultured in a 6-well plate and transfected at a confluency of ∼80 %. 1–2 μg of plasmid DNA was transfected using GoldenTran-DR (Golden Transfer Technology, China). RNAi experiments were performed as described previously [32]. All siRNAs were obtained from RiboBio or Gene Pharma (China) with sequences listed in Suppl. Table 1.

### 2.3 Stable cell lines

For MEG3 overexpression in MCF7 and MDA-MB-231 cell lines, pLVX-TRE3G and pLVX-TET3G doxycycline (DOX) inducible system was used. For MEG3 knockdown in MCF10A with CRISPRi, pSLQ1651-sgTelomere(F + E) and pHR-SFFV-dCas9-BFP-KRAB were used. sgRNA core sequences were listed in Suppl. Table 1. These plasmids were packed with virus proteins in 293T cell line, collected after 48 h, and used to infect the desired cell line followed by puromycin selection.

### 2.4 RNA fluorescence in situ hybridization (RNA-FISH)

RNA-FISH was conducted as previously described [32]. Briefly, cells were fixed with 4 % paraformaldehyde 24 h after transfection, and permeabilized using 0.1 % Triton X-100. Following washing in 1 × SSC/50 % formamide, the cells were subjected to overnight probe incubation at 37 °C. Nuclear staining was achieved using DAPI. Fluorescence signals were visualized using a DMI8 microscope (Leica).

### 2.5 Preparation of nuclear extract

The nuclear/cytoplasmic separation was done as described [33]. Briefly, HeLa or MDA-MB-231 cell lines were detached at 95 % confluency using a cell scraper and treated with a hypotonic buffer. The cells were then lysed using a Dounce homogenizer. The nuclear fraction was extracted using low- and high-salt buffers, followed by dialysis in a buffer (20 mM HEPES, 100 mM KCl, 0.2 mM EDTA, and 20 % glycerol) using MINI dialysis units (Thermo).

### 2.6 RNA-seq

Four cell lines (MEG3-KD and MCF10A control, MEG3-OE and MCF7 control) were subjected to total RNA extraction using TRIzol, RNA-seq analyses were performed using Illumina Hiseq X Ten platform by Novogene company (China).

### 2.7 Purification of MEG3 lncRNPs assembled in vivo

Affinity purification of MEG3 lncRNPs formed *in vivo* was performed as previously described [31]. Briefly, MEG3 cDNA was cloned into a pcDNA5/FRT/TO vector containing MS2 hairpins. This construct was co-transfected with a vector encoding OG44 into FRT-HeLa cells to establish an inducible stable cell line. Nuclear extracts were prepared after DOX induction, and MEG3 RNPs assembled *in vivo* were purified using MS2-MBP affinity purification. The antisense sequence of MEG3 was utilized as a negative control. Purified lncRNPs were separated on SDS-PAGE and visualized by silver staining. Proteins in the lncRNPs were determined using mass spectrometry analysis performed at the core facility of Tsinghua University.

### 2.8 RNA immunoprecipitation (RIP)

RNA Immunoprecipitation was performed as described previously [32]. Briefly, protein A agarose beads (GE Healthcare) coupled with an anti-CTCF antibody (IgG antibody as a control) were used for RIP in a 100 μL binding buffer (20 mM HEPES, pH 7.9, 500 mM KCl, 0.1 % Triton X-100, 0.25 mM EDTA). The immunoprecipitated complexes were incubated with rotation at 4 °C for 2 h and washed six times with 1 mL of binding buffer. Protein digestion was performed using 10 mg/mL Proteinase K (Merck) at 37 °C for 10 min. RNA was purified using phenol/chloroform extraction and ethanol precipitation. The isolated RNAs were reverse transcribed and subjected to PCR analysis using primers listed in Suppl. Table 2.

### 2.9 Statistics

Data are presented as mean ± standard deviation (S.D.). Statistical comparisons between two groups were conducted using Student's *t*-test, with statistical significance indicated by ∗p < 0.05, ∗∗p < 0.01, and ∗∗∗p < 0.001.

## 3 Results

### 3.1 Decreased expression of nuclear lncRNA MEG3 in breast cancer

**Fig. 1:**
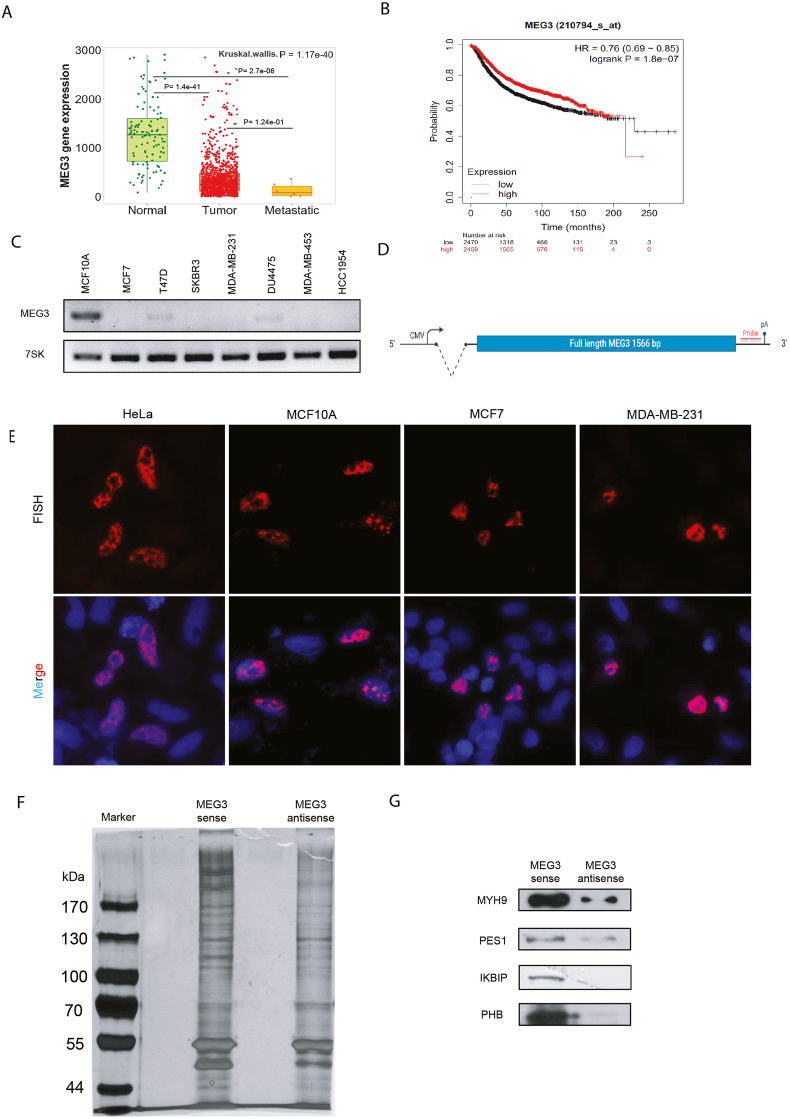
Decreased expression of nuclear lncRNA MEG3 in breast cancer. (A) Detection of MEG3 in normal, tumor, and metastatic breast cancer in TNMplot database. (B) Kaplan-Meier recurrence-free survival analysis of MEG3 using breast cancer data in Kaplan-Meier Plotter database. (C) RT-PCR of endogenous lncRNA MEG3 expression in various breast cancer cell lines. (D) Schematic of the MEG3 construct, β-globulin intron showed as dotted line, numbers represent the length of MEG3 transcript, cDNA showed as box, pA (poly A signal), probe showed as red hybridized sequence. (E) RNA-FISH to visualize the localization of MEG3 transcript post-transfection in HeLa, MCF10A, MCF7 and MDA-MB-231. DAPI staining was utilized to identify the nucleus. (F) Silver stain for lncRNPs assembled on the sense strand of MEG3 with the antisense strand used as a control, separated on SDS-PAGE. (G) Western blot analysis for the selected proteins for MEG3 mass spectrometry data verification.

Previous reports suggested that MEG3 was a nuclear lncRNA with decreased expression in many different malignancies including breast cancer. Here we first verified the expression level of MEG3 in breast cancer using TNM plot database [34]. As compared to the normal tissues, the expression level of MEG3 was significantly downregulated in breast cancer (p = 1.17e-40) (Fig. 1A). Specifically, a substantial decrease of MEG3 expression was observed in tumor samples (p = 1.4e-41), with the lowest expression observed in metastatic samples (p = 2.7e-06) (Fig. 1A). Furthermore, lower expression of MEG3 was correlated to poor prognosis when the recurrence-free survival of breast cancer was assayed using Kaplan-Meier Plotter database [35] (HR = 0.76 (0.69–0.85), p = 1.8e-07) (Fig. 1B).

Moreover, we checked the expression of MEG3 in a subset of breast cancer cell lines, the results indicated that only the human normal breast cell line MCF10A showed a high expression of MEG3, whereas remarkably decreased expression of MEG3 was observed in all the 7 breast cancer cell lines including MCF7, T47D, SKBR3, MDA-MB-231, DU4475, MDA-MB-453 and HCC1954 (Fig. 1C).

Subsequently, we employed lncRNA MEG3 reporter with an optimized β-globin intron 1 inserted upstream of MEG3 cDNA under CMV promoter [2] (Fig. 1D). After transient transfection of MEG3 reporter in HeLa, MCF10A, MCF7, and MDA-MB-231, RNA-FISH was conducted to detect the localization of lncRNA MEG3 transcripts and MEG3 transcripts displayed a dominating nuclear localization in all used cell lines (Fig. 1E). Consistent with previous reports, our results confirmed that MEG3 was a nuclear lncRNA with reduced expression in breast cancer cell lines.

### 3.2 Purification of MEG3 lncRNP formed in vivo

**Supplementary Fig. 1:**
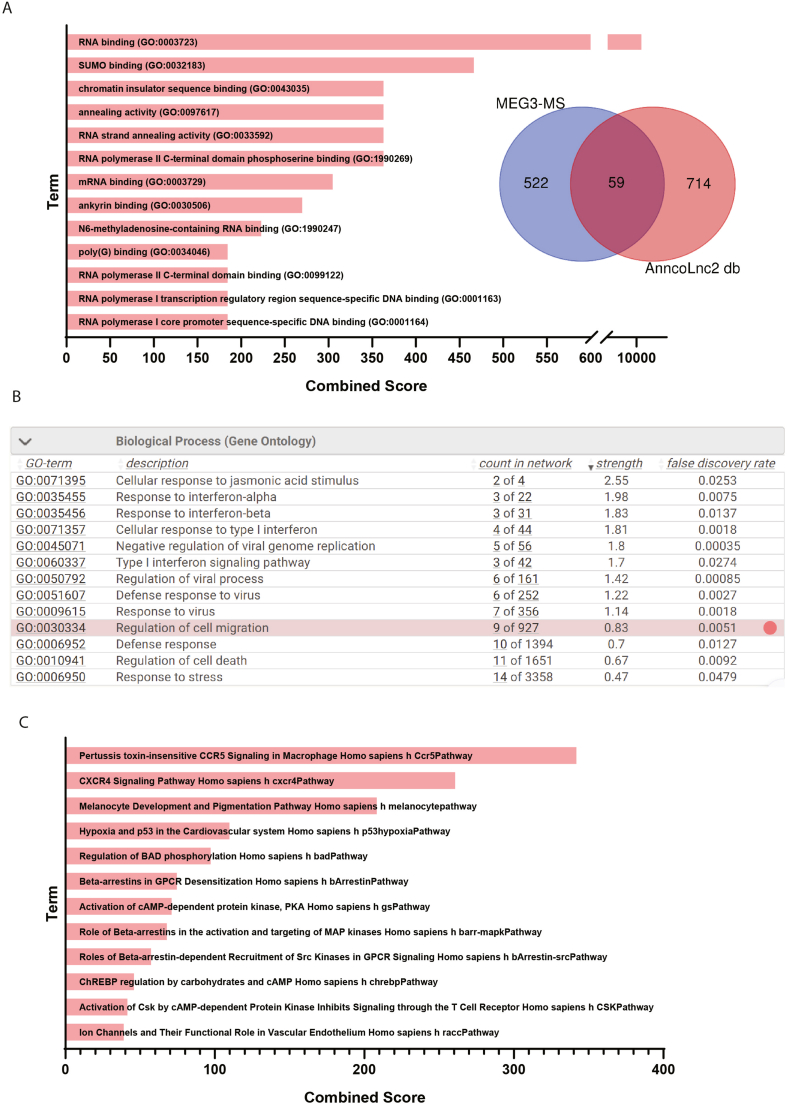
GO analysis for MEG3 mass spectrometry and RNA seq data (A) Gene ontology analysis (Molecular function) based on Enrichr database for our MEG3 mass spectrometry data overlapped with the predicted MEG3-binding proteins using Annolnc2. (B) Gene ontology analysis (Biological process) for the differentially expressed genes with a log2 fold change more than −1.5 and 1.5 and a significance level of p_adj_ < 0.05 using String database. (B) Pathway analysis for the differentially expressed genes using Enrich database.

To identify proteins bound to MEG3 which may mediate MEG3 function in tumor progression, we attempted to purify MEG3 lncRNP assembled *in vivo*. Silver staining showed distinct patterns of proteins in MEG3 lncRNP vs lncRNP formed on the antisense of MEG3 after separation on SDS-PAGE (Fig. 1F). Proteins in the lncRNPs were assayed by mass spectrometry, which revealed a total of 593 proteins enriched in MEG3 lncRNP (Suppl. Table 3). Next, Western blot analysis of selected proteins of different sizes confirmed the enrichment of these proteins on the sense strand compared to the antisense (Fig. 1G). Proteins from mass spectrometry data were overlapped with prediction obtained from AnnoLnc2 database [36], which revealed 59 shared proteins. GO analysis of the overlapped MEG3 binding protein using Enrichr database [37] revealed the association of MEG3 binding proteins with various GO terms such as RNA binding, SUMO binding, and chromatin insulator sequence binding (Suppl. Fig. 1A).

### 3.3 MEG3 is crucial for breast cancer cell proliferation and migration

**Fig. 2:**
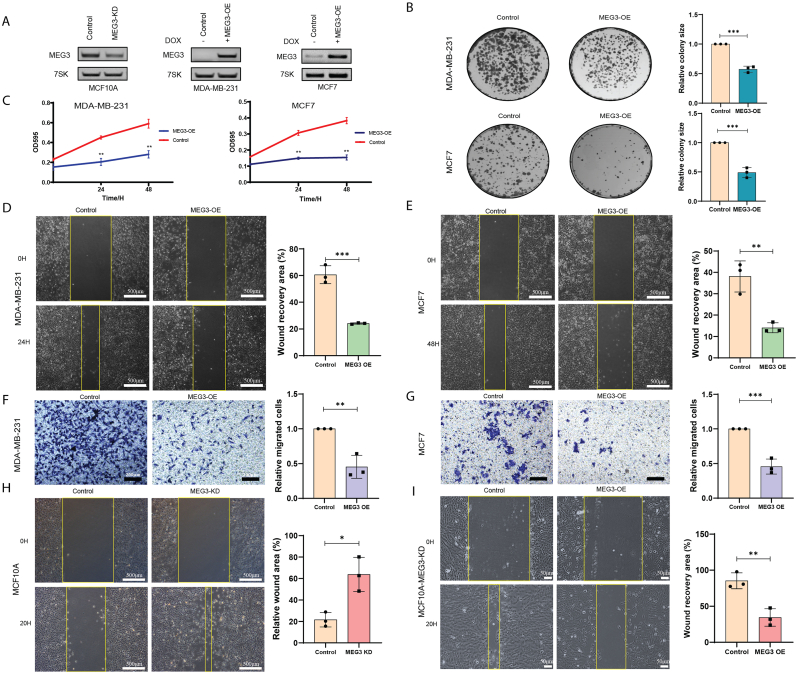
MEG3 is crucial for breast cancer cell proliferation and migration. (A) RT-PCR analysis of MEG3 expression in MEG3-KD cell line (MCF10A), MEG3-OE cell lines (MDA-MB-231, and MCF7), respectively. KD: knockdown, OE: overexpression. (B) Colony formation assay to assess the impact of MEG3 overexpression in MDA-MB-231 and MCF7 cell lines (n = 3, ∗∗∗p < 0.001). (C) MTT analysis of cell viability in MEG3-OE cell lines MDA-MB-231 and MCF7 (n = 3, ∗∗p < 0.01). (D–E) Wound healing assay to demonstrate the migratory capacity of MDA-MB-231 and MCF7 cell lines following MEG3 overexpression (the scale bar represents 500 μm) and further evaluated using a Transwell assay (the scale bar represents 200 μm) (F–G) (n = 3, ∗∗p < 0.01, ∗∗∗p < 0.001). (H) Wound healing assay in MCF10A-MEG3-KD cell line (n = 3, ∗p < 0.05) (the scale bar represents 500 μm). (I) Wound healing assay in MCF10A-MEG3-KD cell line following re-expression of MEG3 (the scale bar represents 50 μm) (n = 3, ∗∗p < 0.01).

To investigate the potential role of MEG3 in breast cancer progression, we first established three stable cell lines including MEG3 knockdown cell line using CRISPRi technique in MCF10A, and inducible MEG3 overexpression in two commonly used breast cancer cell lines MDA-MB-231 (TNBC) and MCF7 (Luminal). RT-PCR assay showed the knockdown efficiency in MCF10A and increased level of MEG3 in MDA-MB-231 and MCF7 cell lines after DOX induction for 24 h (Fig. 2A). Next, we performed clone formation and MTT assay to determine the impact of MEG3 on breast cancer cell proliferation. We observed a significant reduction in colony size in both cell lines upon MEG3 overexpression (Fig. 2B) as well as a strong inhibition on cell proliferation (Fig. 2C). Furthermore, we performed wound-healing and Transwell assay to explore the role of MEG3 on cell migration. Remarkably, MEG3 overexpression in both cell lines led to significantly reduced wound-healing (Fig. 2D and E) and decreased number of migrated cells (Fig. 2F and G). There is no noticeable variation in the response to MEG3 overexpression between the two cell lines. Lastly, to further build up the correlation between the MEG3 expression level and breast cancer cell migration, we performed wound-healing assay on the MEG3-depleted MCF10A cells. Most strikingly, MEG3 depletion in MCF10A resulted in prominent increase in cell migration (Fig. 2H), whereas re-introduction of MEG3 in this cell line using transient transfection partially restored the inhibition of cell migration capacity (Fig. 2I). These results altogether suggested that the expression level of MEG3 was vital to breast cancer cell proliferation and migration.

### 3.4 CXCR4 is the major downstream mediator regulated by MEG3

**Fig. 3:**
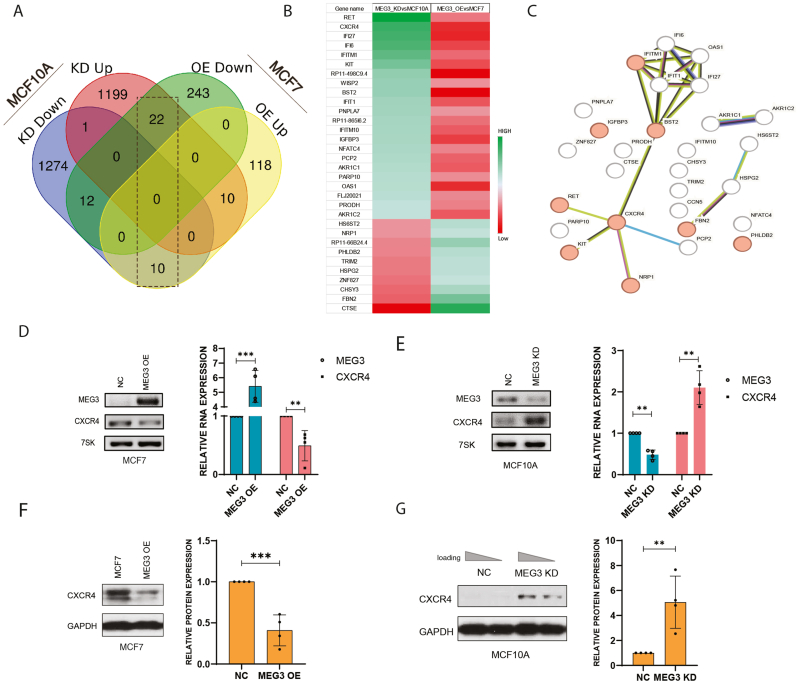
CXCR4 is the major downstream mediator regulated by MEG3. (A) Venn diagram illustrating the overlap of differentially expressed genes upon MEG3 knockdown and overexpression. (B) Heatmap representation of the differentially expressed genes with a log2 fold change more than −1.5 and 1.5 and a significance level of padj<0.05. (C) Proteins association network analysis using String database. (D–E) RT-PCR analysis showing the expression of CXCR4 at RNA level upon MEG3 knockdown or overexpression (n = 4, ∗∗p < 0.01, ∗∗∗p < 0.001). (F–G) Western blot analysis showing the expression of CXCR4 at protein level upon MEG3 knockdown or overexpression (n = 4, ∗∗p < 0.01, ∗∗∗p < 0.001).

Since cell migration turned out to be the most affected phenotype upon MEG3 depletion or upregulation, we aimed to identify the major downstream mediator for this change. RNA-sequencing on four cell lines (MCF10A, MCF10A with MEG3 knockdown, MCF7 and MCF7 with MEG3 overexpression) was performed and the differentially expressed genes were filtered by the strength of log2 fold expression levels with a cut-off point at 1.5 or −1.5 (p_adj_ < 0.05). The analysis revealed a total of 1232 genes upregulated and 1297 genes downregulated upon MEG3 depletion in MCF10A as well as 138 genes upregulated and 277 genes downregulated upon overexpression in MCF7 (Suppl. Table S4). Overlapping these genes using the Venn diagram resulted in 32 shared genes (Fig. 3A and B), which are potential candidates of downstream targets regulated by MEG3. GO analysis of Biological Process highlighted "Regulation of cell migration" as one of the top biological processes affected by the knockdown or overexpression of MEG3 (Suppl. Fig. 1B). Pathway analysis using Enrichr database further revealed CXCR4 signaling pathway as one of the top most affected pathways (Suppl. Fig. 1C). Additionally, STRING database was used to draw the functional protein association network (Fig. 3C), which illustrated CXCR4 as one of the connecting protein nodes that function in cell migration. These analyses together suggested CXCR4 as a strong downstream target regulated by MEG3 which might have a direct impact on cell migration.

To test whether CXCR4 is a major downstream target regulated by MEG3, RT-PCR was performed using total RNAs from MEG3-OE MCF7 and MEG3-KD MCF10A. MEG3 overexpression in MCF7 led to substantially decreased expression of CXCR4 at RNA level (Fig. 3D), whereas MEG3 depletion in MCF10A resulted in enhanced CXCR4 expression at RNA level (Fig. 3E). Consistent to the RT-PCR results, Western blot analysis on both cell lines indicated reduced CXCR4 expression at protein level upon MEG3 overexpression in MCF7 (Fig. 3F) and elevated CXCR4 expression at protein level upon MEG3 knockdown in MCF10A (Fig. 3G). These results together supported that CXCR4 was the major downstream target negatively regulated by MEG3.

### 3.5 MEG3 regulates cell migration via CXCR4

**Fig. 4:**
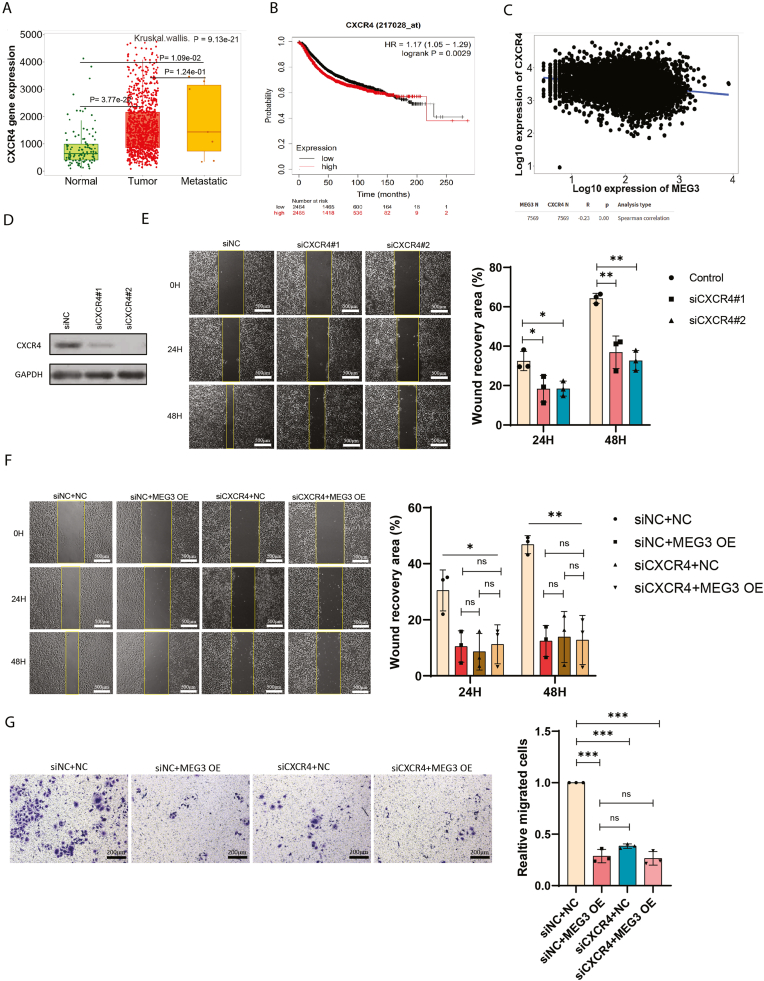
MEG3 regulates cell migration via CXCR4. (A) Expression of CXCR4 in normal, tumor, and metastatic breast cancer from TNMplot database. (B) Kaplan-Meier recurrence-free survival analysis for CXCR4 using breast cancer data in Kaplan-Meier Plotter database. (C) Spearman correlation analysis between MEG3 and CXCR4 in breast cancer data from TNMplot database. (D) Western blot analysis of CXCR4 knockdown using two siRNAs. (E) Wound healing assay for MCF7 following CXCR4 knockdown using two siRNAs (n = 3, ∗p < 0.05, ∗∗p < 0.01) (the scale bar represents 500 μm). (F–G) Wound healing and Transwell assay for MEG3 re-expression in MCF7 after depletion of CXCR4 (n = 3, ∗p < 0.05, ∗∗p < 0.01, ∗∗∗p < 0.001, ns: no significance) (the scale bar represents 500 μm for Wound healing assay and 200 μm for Transwell assay).

**Supplementary Fig. 2:**
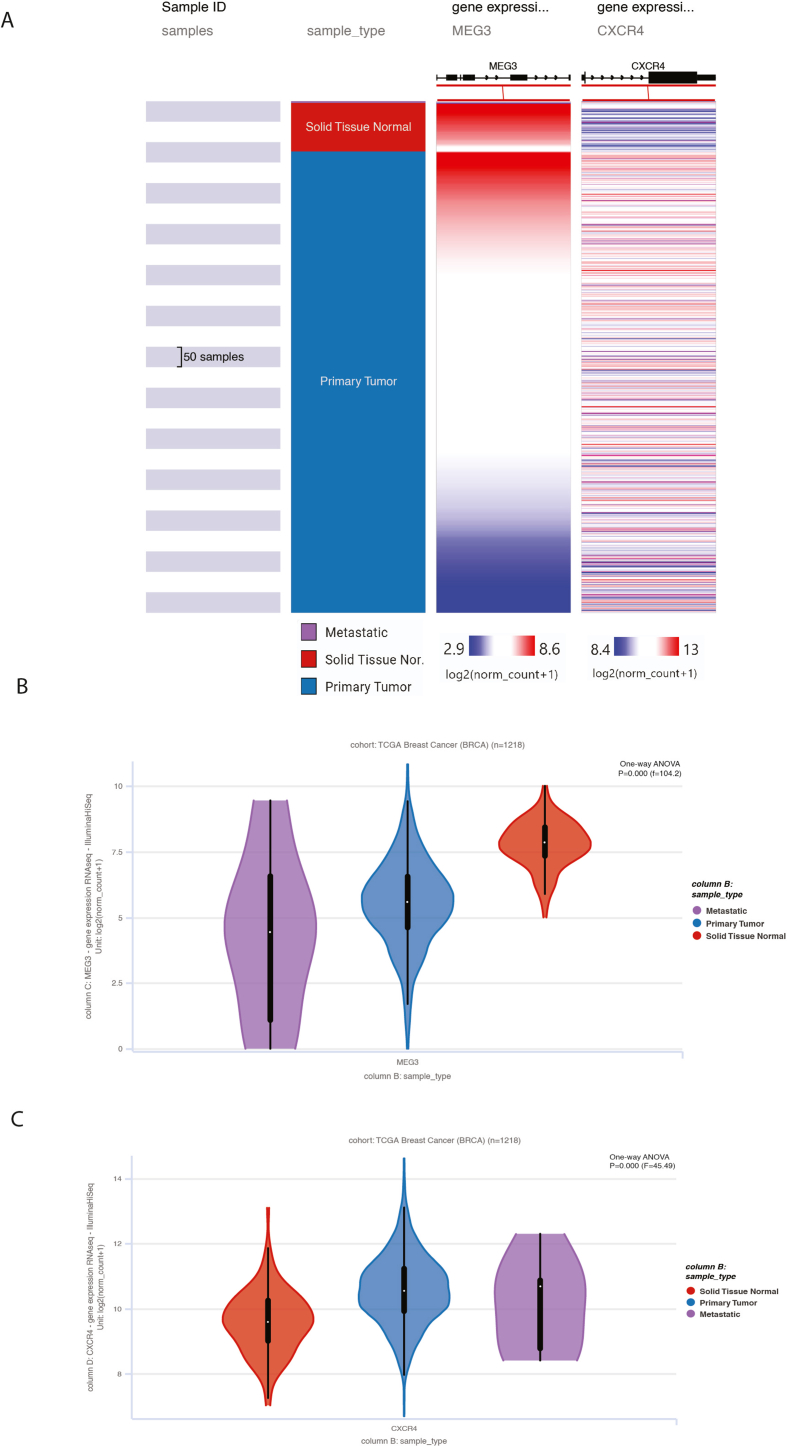
RNA seq data analysis for TCGA breast cancer using UCSC Xena Database (A) Heatmap of MEG3 and CXCR4 expression in TCGA breast cancer data (n = 1218) according to sample type from UCSC Xena database. (B) Violin view of MEG3 expression in TCGA breast cancer data. (C) Violin view of CXCR4 expression in TCGA breast cancer data.

Interestingly, bioinformatic analysis on CXCR4 expression in breast cancer using TNM plot database [34] revealed that CXCR4 was upregulated in breast cancer samples as compared to normal breast tissues (p = 3.77e-22, Fig. 4A). Recurrence-free survival analysis for CXCR4 using breast cancer data in Kaplan-Meier Plotter Database [35] showed that the upregulation of CXCR4 was associated with poor prognosis (HR = 1.17 (1.05–1.29), p = 0.0029, Fig. 4B). This result was further confirmed using UCSC Xena database [38] which showed decrease of MEG3 expression in primary and metastatic tumor compared to normal solid tissue (Suppl. Fig. 2A and B), whereas CXCR4 expression was found to be upregulated in primary and metastatic tumor (Suppl. Fig. 2A and C). Furthermore, TNMplot database [34] also pinpointed a negative correlation between MEG3 and CXCR4 expression in breast tumor samples (Fig. 4C).

In order to evaluate the impact of CXCR4 on the migration of breast cancer cells, we used two siRNAs to knockdown CXCR4 in MCF7 respectively, the knockdown efficiency was confirmed in Fig. 4D by Western blot. As shown in Fig. 4E, knockdown of CXCR4 using either siRNA led to significant inhibition on cell migration as assayed by wound healing at either 24 h or 48 h. To further determine the role of CXCR4 in MEG3-mediated cell migration, we re-expressed MEG3 in MCF7 with CXCR4 depletion. Intriguingly, restoring MEG3 level in MCF7 led to inhibition of cell migration, as a sharp contrast, no change in the inhibition of cell migration was observed when MEG3 was restored in MCF7 with CXCR4 depletion (Fig. 4F and G). Altogether, these results indicated that CXCR4 was the major target downstream MEG3 and MEG3 regulated breast cancer cell migration via CXCR4.

### 3.6 MEG3 interacts with CTCF to restrain the transcriptional activation on CXCR4

**Fig. 5:**
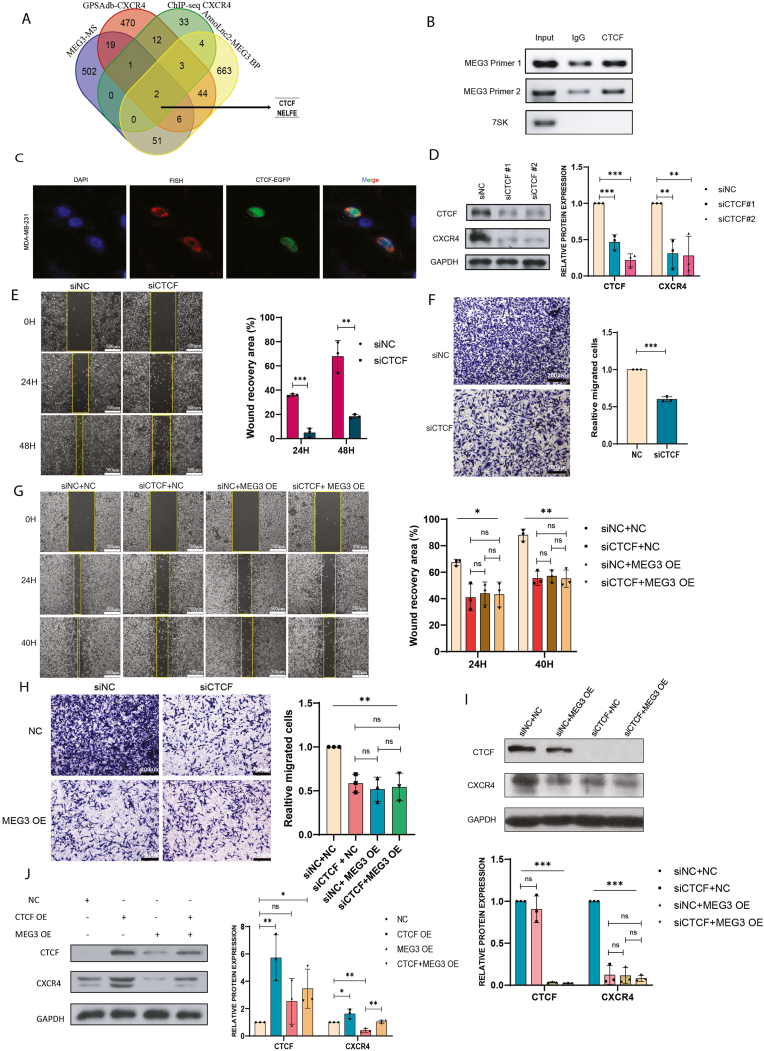
MEG3 interacts with CTCF to restrain the transcriptional activation on CXCR4. (A) Venn diagram illustrates the proteins detected in our MEG3 mass spectrometry data, predicted MEG3 binding proteins from the AnnoLnc2 database, CXCR4-ChIP-seq data from the Cistrome toolkit database, and data from genetic perturbation similarity analysis for CXCR4 upstream regulators from the GPSAdb (Duplicated gene names were removed). (B) RT-PCR was performed to analyze MEG3 lncRNA precipitated from RIP experiment with a CTCF antibody, IgG antibody was used as a control. (C) RNA-FISH was used to demonstrate the co-localization of the full-length MEG3 transcript and the CTCF-EGFP protein. (D) Western blot analysis was conducted on MDA-MB-231 cells following the knockdown of CTCF using two siRNAs to assess the impact on CXCR4 expression (n = 3, ∗∗p < 0.01, ∗∗∗p < 0.001). (E) Wound healing assay to evaluate the migratory capacity of MDA-MB-231 cells after the downregulation of CXCR4 caused by CTCF knockdown (n = 3, ∗∗p < 0.01, ∗∗∗p < 0.001) (the scale bar represents 500 μm). (F) Transwell assay for MDA-MB-231 after CTCF depletion (n = 3, ∗∗∗p < 0.001) (the scale bar represents 200 μm). (G&H) Wound healing assay and Transwell assay for MEG3 overexpression and CTCF depletion dual treatment (n = 3, ∗∗p < 0.01, ∗∗∗p < 0.001, ns: no significance) (the scale bar represents 500 μm for Wound healing assay and 200 μm for Transwell assay). (I) Western blot analysis showing the effect of MEG3 overexpression in combination with CTCF depletion on CXCR4 gene expression (n = 3, ∗∗∗p < 0.001, ns: no significance). (J) Western blot analysis showing the effect of dual overexpression of MEG3 and CTCF on CXCR4 gene expression (n = 3, ∗p < 0.05, ∗∗p < 0.01, ns: no significance).

To identify potential candidate MEG3 binding proteins mediating CXCR4 expression, we overlapped the MEG3 binding proteins generated from our MEG3 mass spectrometry data, the predicted MEG3 binding proteins from the AnnoLnc2 database [36], CXCR4-ChIP-seq data from the Cistrome toolkit database [39], and data from genetic perturbation similarity analysis for CXCR4 upstream regulators from the GPSAdb [40], the overlapped results revealed two proteins (CTCF and NELFE) that could bind with MEG3 and regulate CXCR4 (Fig. 5A), with CTCF exhibiting higher fold enrichment compared to NELFE. To verify the binding of CTCF to MEG3, we conducted RIP and the results revealed the enrichment of MEG3 with the CTCF antibody (Fig. 5B). Furthermore, we determined the localization of exogenous overexpressed MEG3 transcripts and CTCF proteins in MDA-MB-231 cell line, which revealed partial co-localization of MEG3 transcripts and CTCF (Fig. 5C).

To investigate the impact of CTCF on CXCR4 expression, two siRNAs were used to knockdown CTCF in MDA-MB-231 cells. As shown in Fig. 5D, depletion of CTCF led to significantly lower expression of CXCR4. Next, to assess the impact of CXCR4 downregulation upon CTCF depletion on cell migration, we conducted wound healing assay and Transwell assay in MDA-MB-231. The results showed a significant reduction in cell migration due to CTCF knockdown (Fig. 5E and F). Furthermore, to investigate the potential role of MEG3-CTCF interaction on CXCR4 gene expression and cell migration, we overexpressed MEG3 in MDA-MB-231 cells in combination with CTCF depletion. As shown in Fig. 5G and H, the combined effects of MEG3 overexpression and CTCF knockdown led to a migration inhibition effect similar to that seen with MEG3 overexpression or CTCF knockdown alone. Furthermore, the downregulation of CXCR4 expression due to the combined influence of MEG3 overexpression and CTCF depletion reflected the same level of downregulation achieved by MEG3 overexpression alone (Fig. 5I). In addition, we performed a dual overexpression of CTCF and MEG3. As shown in Fig. 5J, CTCF overexpression led to enhanced expression of CXCR4, whereas co-expression of CTCF and MEG3 abated CXCR4 expression. Collectively, these data indicate that MEG3 interacts with CTCF to restrain its transcriptional activation on CXCR4.

## 4 Discussion

LncRNAs function in different biological processes and cellular pathways with disrupted expression of lncRNAs reported in many diseases. Among them, expression of MEG3 is frequently lost/decreased in different malignancies and accumulated evidence supports that MEG3 functions as a tumor suppressor.

In this study, we first verified the decreased expression of MEG3 in breast cancer samples using bioinformatic analysis on TNM plot database as well as in breast cancer cell lines using RT-PCR. We provided further evidence to show that cell migration was the phenotype with close correlation to MEG3 expression level in breast cancer cell lines. MEG3 overexpression significantly inhibited migration in two breast cancer cell lines MCF7 (Luminal) and MDA-MB-231 (TNBC), but no noticeable differences were found between the two cell lines, indicating the effect of MEG3 on migration independent on the subtype of breast cancer. Next, extensive bioinformatic analyses on RNA-seq data from MCF7 with MEG3 overexpression and MCF10A with MEG3 depletion suggested CXCR4 as the major target downstream MEG3 that mediated breast cancer cell migration. Experimental data further verified that the expression of CXCR4 was negatively correlated with MEG3 expression level. Whereas depletion of CXCR4 in breast cancer cells resulted in substantial suppression of cell migration. Interestingly, several previous studies reported CXCR4 as one of the key receptors on the cell membrane that controlled breast cancer cell migration [[41], [42], [43]]. The alpha-chemokine receptor CXCR4, also referred to as fusin or CD184, is specific for stromal-derived factor-1 (SDF-1, also known as CXCL12). CXCR4 is a transmembrane domain heterotrimeric G protein-coupled receptor superfamily member that is expressed functionally on the surface of different kinds of cancer cells. It has recently emerged that CXCR4 is crucial for the survival, proliferation, migration, and metastasis of various cancers, including breast cancer[[44], [45], [46]]. CXCR4 signaling promoted chemotactic and invasive features by facilitating actin polymerization and pseudopodia development [47]. *In vivo*, suppressing CXCL12/CXCR4 interactions drastically decreased breast cancer cell metastasis to the surrounding lymph nodes and lungs [48].

It is well known that RNA-protein interactions are vital for RNA to function. A recent review on lncRNA-protein interactions further fortifies the concept of proteins bound on lncRNAs in mediating the role of different lncRNAs. To find out how MEG3 regulates CXCR4 gene expression, we purified MEG3 lncRNP formed *in vivo* with the protocol established in the lab [31] and mass spectrometry analysis revealed ∼600 proteins presented in MEG3 lncRNP. Among them, CTCF and NELFE emerged as potential candidate regulators on CXCR4 expression after comparison of proteins in MEG3 lncRNP to MEG3-binding proteins predicted with the AnnoLnc2 database as well as CXCR4-ChIP-seq data from the Cistrome database and upstream regulators from GPSAdb database for CXCR4 gene. We focused on CTCF in this study due to its higher enrichment. CTCF or CCCTC-binding factor is a zinc finger protein containing eleven tandem zinc finger DNA-binding domain [49] that is highly conserved and involved in various regulatory functions, such as transcriptional activation/repression, insulation, imprinting, and X chromosome inactivation [50,51]. CTCF can bind to chromatin domain boundaries, enhancers, gene promoters, and within gene bodies and attract transcription machinery including RNA polymerase II, cohesin, and tissue-specific activators and repressors to chromatin to form chromatin loops [52]. CTCF has also been reported to play a crucial role in EMT/MET by regulating the expression of E-cadherin and N-cadherin, and diminished enrichment of CTCF binding motifs was shown to be one of the key features in the early phase of EMT as assayed using ATAC-seq for chromatin accessibility [53].

A previous study showed that chromatin regulator CTCF functioned as an RNA-binding protein and it could be displaced from the promoter of lncRNA Xist by Jpx RNA [54]. Multiple regions within CTCF have been shown to interact with RNA [[55], [56], [57]]. Interestingly, ncRNA binding to CTCF was shown to impair its DNA-binding and activate the expression of target genes [58]. In pancreatic cancer, binding of CTCF to lncRNA PACERR was reported to increase histone acetylation, which enhanced transcription and promoted M2 polarization of tumor-associated macrophage [59]. Moreover, binding of CTCF to lncRNA HOTTIP was reported to promote EFNA3 transcription by recruiting CTCF to the EFNA3 promoter [60].

**Fig. 6:**
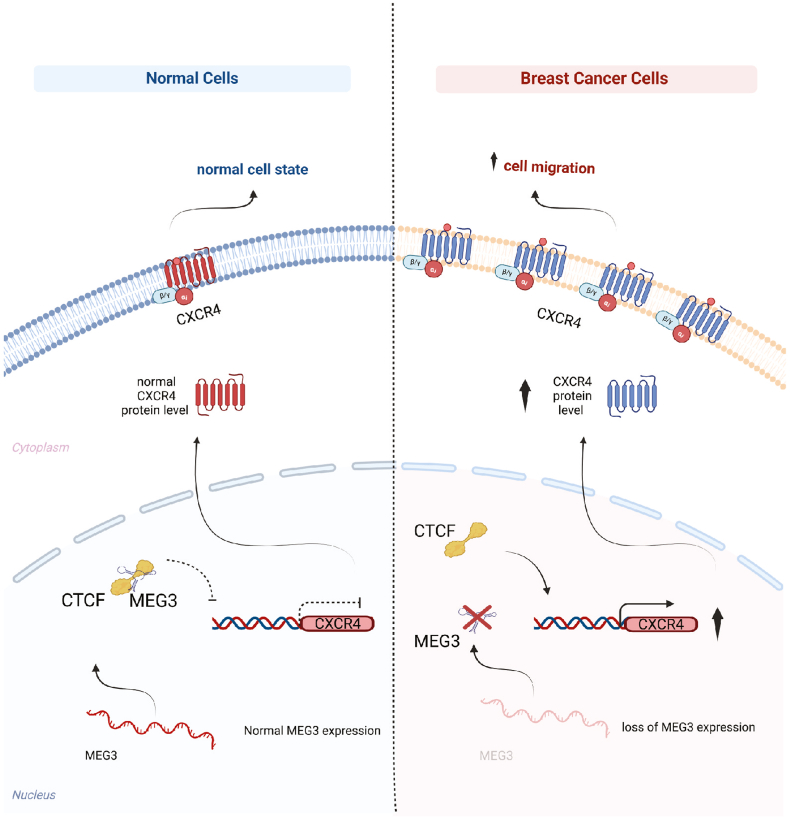
MEG3/CTCF-CXCR4 axis regulates breast cancer cell migration. Study model: The expression of lncRNA MEG3 in the nucleus of normal cells allows interaction of CTCF protein to MEG3, which restrains CTCF from activating CXCR4 transcription. Consequently, CXCR4 is expressed at a low level that cannot promote cell migration. In contrast, since the expression of MEG3 is lost or significantly reduced in breast cancer cells, CTCF acts freely to activate the expression of CXCR4. Breast cancer cells with a high level of CXCR4 are subject to enhanced cell migration. Created with BioRender.com.

Our study together with these reports supported the model of MEG3/CTCF-CXCR4 axis in the cell migration of breast cancer development, in which CTCF serves as a transcription activator. As illustrated in Fig. 6, expression of lncRNA MEG3 in the nucleus of normal cells allows interaction of CTCF protein to MEG3, which restrains CTCF from binding to the promoter/enhancer region of CXCR4. Thus, CXCR4 is expressed at a low level that cannot promote cell migration. In contrast, since the expression of MEG3 is lost or significantly reduced in breast cancer cells, CTCF is recruited to the promoter/enhancer region of CXCR4 to promote the transcription. Breast cancer cells with a high level of CXCR4 are subject to enhanced cell migration.

Intriguingly, CTCF interacting protein nucleoporin 153 [61] and condensin complex subunits SMC4 and SMC2 [62] served as negative regulators of CTCF were all enriched in the MEG3 lncRNP, further investigation on the interactions between MEG3 and these factors will help to address the detailed mechanism of MEG3 in cancer progression by regulating downstream target genes.

## CRediT authorship contribution statement

**Gusai Elhassan:** Writing – original draft, Visualization, Validation, Software, Methodology, Investigation, Formal analysis, Data curation, Conceptualization. **Xiangxue Bu:** Methodology, Investigation, Data curation, Conceptualization. **Jiaxin Liu:** Visualization, Software, Resources, Project administration, Investigation. **Shuai Hou:** Writing – review & editing, Visualization, Validation, Supervision, Resources, Project administration, Methodology, Investigation, Funding acquisition, Data curation, Conceptualization. **Jinsong Yan:** Writing – review & editing, Supervision, Project administration, Methodology, Conceptualization. **Haixin Lei:** Writing – review & editing, Validation, Supervision, Resources, Project administration, Investigation, Funding acquisition, Conceptualization.

## Availability of data

The data that support this study are available from the corresponding author upon request.

## Declaration of competing interest

The authors declare that they have no known competing financial interests or personal relationships that could have appeared to influence the work reported in this paper.
