# Supplementary material for: LncRNA MEG3/CTCF-CXCR4 axis functions in the regulation of breast cancer cell migration
Source: Noncoding RNA Res. 2025 May 28;14:117–28. doi: 10.1016/j.ncrna.2025.05.014 (PMC12178829; doi:10.1016/j.ncrna.2025.05.014)
Supplement: Multimedia component 5 [file mmc5.docx]

**Supplementary material and methods**

**MTT**

After preparing cell suspension, 3000-5000 cells per well were cultured in a 96-well plate (or 6000-10000 cells per well in a 48-well plate). 200 μL of medium was added to each well. After DOX induction, time points were established, and 10% MTT solution was added to each well at a final concentration of 0.5 mg/mL. After 4 hours of incubation, the solution was removed, and 150 μL of dimethyl sulfoxide was added to each well. The plate was gently shaken in the dark for 10 minutes and measured using microplate reader (EnSpire Multimode Plate Reader, PerkinElmer) at the absorbance of 595 nm.

**Colony formation assay**

A single-cell suspension was prepared from well-growing cells and counted, 1000-2000 cells per well were cultured in a 6-well plate and incubated at 37°C for 1-2 weeks, with the medium being changed every three days during the incubation period with the addition of the drug (DOX). After the incubation period, the cells were fixed with 4% paraformaldehyde for 15 min followed by washing with PBS. Then, stained with 0.1% crystal violet for 10 minutes, followed by a gentle wash with water to remove the staining solution. The clone size area was calculated using imageJ software to determine the relative colony size.

**Wound healing assay**

After culturing the cells in flat-bottomed 6 well plate, a scratch was made using a yellow tip (200 μL), the cells were then washed with PBS to remove non-adherent cells. Low-serum medium was added based on the experimental requirements. 0-hour picture was taken immediately after wound creation other pictures were taken according to the indicated time points using DMI4000 B (Leica) or ICX41 (SOPTOP). The plates were placed in a 37°C, 5% CO_2_ cell culture incubator. The migration area was measured and quantified using ImageJ and calculated to determine the wound recovery rate.

**Transwell assay**

After preparing cell suspension, 1.2 x10^5^ – 2 x10^5^ cells were cultured into the upper chamber of transwell champers with serum-free medium and 10-15% FBS medium in the lower chamber. Following a predetermined incubation period, non-migratory cells present on the upper side of the membrane were carefully removed, while the migrated cells on the lower side were fixed with 4% paraformaldehyde for 15 min. Subsequently, the fixed cells were washed with PBS and stained with a 0.1% crystal violet solution for 15 min. After staining, the excess stain was washed away gently with PBS. The pictures were taken using DMI4000 B (Leica). The migrated cells area was then quantified by ImageJ software and calculated to determine the relative migrated cells.
